# Towards robust probabilistic maps in Deep Brain Stimulation: exploring the impact of patient number, stimulation counts, and statistical approaches

**DOI:** 10.3389/fncom.2025.1699192

**Published:** 2026-01-21

**Authors:** Vittoria Bucciarelli, Dorian Vogel, Karin Wårdell, Jérôme Coste, Patric Blomstedt, Jean-Jacques Lemaire, Raphael Guzman, Simone Hemm, Teresa Nordin

**Affiliations:** 1 Institute for Medical Engineering and Medical Informatics, School of Life Sciences, University of Applied Sciences and Arts Northwestern Switzerland FHNW, Muttenz, Switzerland; 2 Department of Biomedical Engineering, University of Basel, Allschwil, Switzerland; 3 Department of Biomedical Engineering, Linköping University, Linköping, Sweden; 4 Institut Pascal, CNRS, CHU Clermont-Ferrand, Clermont Auvergne INP, Université Clermont Auvergne, Clermont-Ferrand, France; 5 Department of Clinical Science, Neuroscience, Umeå University, Umeå, Sweden; 6 Department of Neurosurgery, University Hospital Basel, Basel, Switzerland

**Keywords:** Deep Brain Stimulation, probabilistic mapping, Probabilistic Sweet Spot, sample size, statistics

## Abstract

**Introduction:**

Probabilistic Stimulation Maps (PSMs) are increasingly employed to identify brain regions associated with optimal therapeutic outcomes in Deep Brain Stimulation (DBS). However, their reliability and generalizability are challenged by the limited size of most patient cohorts and the inherent variability introduced by different statistical methods and input data configurations. This study aimed to investigate the geometrical variability of Probabilistic Sweet Spots (PSS) as a function of both the number of patients (nPat) and the number of stimulations per patient (nStim), and to model a stability boundary defining the minimum data requirements for obtaining geometrically stable PSS.

**Methods:**

Three statistical approaches–Bayesian *t*-test, Wilcoxon test with False Discovery Rate (FDR) correction, and Wilcoxon test with nonparametric permutation correction–were applied to two patient cohorts: a primary cohort of 36 patients undergoing DBS for Parkinson’s Disease (PD), and a secondary cohort of 61 patients treated for Essential Tremor (ET), used to assess generalizability. Stimulation test data was collected intra-operatively for the first cohort and post-operatively for the second one. Geometric stability was evaluated based on variability in PSS volume extent and centroid location.

**Results:**

The analysis revealed a non-linear trade-off between nPat and nStim to yield stable PSS. A stability boundary was defined, representing the minimum combinations of nPat–nStim required for anatomically robust PSS. Among the tested methods, the Bayesian *t*-test achieved stability with smaller sample sizes (∼15 patients) and demonstrated a consistent performance across both cohorts. In contrast, the Wilcoxon-based methods showed variable behavior between cohorts, which differed in symptom type and testing phase (intra-operative testing vs. post-operative screening).

**Discussion:**

The proposed PSS stability boundary provides a practical reference for designing DBS studies and stimulation screening protocols aimed at probabilistic mapping. The Bayesian *t*-test emerged as a reliable method across both cohorts, supporting its potential in studies with limited sample sizes and scenarios where the method needs to be readily generalized to varying symptoms. These findings underscore the importance of considering both cohort size and stimulation count in probabilistic DBS mapping and call for further investigation into method-specific sensitivities to clinical and procedural factors.

## 1 Introduction

Deep Brain Stimulation (DBS) is a well-established neurostimulation method for the alleviation of symptoms of psychiatric and movement disorders such as Parkinson’s Disease (PD), Essential Tremor (ET) and dystonia (Benabid et al., 2000; Wårdell et al., 2022). The therapeutic effect of DBS is achieved by electrically stimulating symptom- and disease-specific deep brain target structures. However, the complexity and partial understanding of the underlying disorder mechanisms pose challenges in defining universally recognized stimulation targets (Lozano et al., 2019). Therefore, increasing focus has been directed toward simulating the extent of the stimulation current spread in the brain tissue to detect the brain areas affected by the stimulation (Hemm-Ode et al., 2016). While the optimal stimulation areas may vary across individuals, group-level analyses performed on patient cohorts can provide valuable insights into average therapeutic targets (Wårdell et al., 2022). Aggregating Volumes of Tissue Activated (VTAs) associated with specific stimulation parameters and their corresponding clinical effects enables the generation of stimulation maps (Eisenstein et al., 2014). Such maps can identify the brain volumes stimulated in the majority of patients (Cheung et al., 2014) or yielding the most favorable therapeutic outcomes (Butson et al., 2010; Shah et al., 2020). Further, the application of statistical analyses at the voxel level within a standardized anatomical space (Vogel et al., 2020) leads to Probabilistic Stimulation Maps (PSMs) (Eisenstein et al., 2014; Dembek et al., 2017; Nowacki et al., 2022; Vogel et al., 2024). PSMs are instrumental in identifying voxels that are significantly associated with symptom improvement. These regions, known as Probabilistic Sweet Spots (PSS), can subsequently inform a range of applications, including models predicting DBS outcomes (Reich et al., 2019; Boutet et al., 2021) and the development of automated approaches for DBS programming (Jaradat et al., 2022; Nordenström et al., 2022). Despite the valuable insights offered by PSMs, some often overlooked drawbacks remain: results are influenced by several parameter and method choices of the probabilistic mapping workflow, such as the chosen statistical or clustering methods (Dembek et al., 2022; Nordin et al., 2022; Bucciarelli et al., 2025a). In addition, given their data-driven nature, the most prominent limitation is the PSMs’ strong dependence on the input data, which results in variable PSS locations and volumes (Kübler et al., 2021; Nordin et al., 2022). This is further exacerbated by the fact that most probabilistic mapping studies are based on small patient cohorts (between 10 and 30 patients), raising concerns about the reliability and generalizability of the findings. Few studies to date have investigated the minimum patient sample size required to achieve PSS that are stable in both anatomical location and volume (Nordin et al., 2023; Bucciarelli et al., 2025b). However, in addition to sample size, the number of available stimulations (e.g., VTAs) must also be considered as a relevant factor. Accordingly, the objective of this study was to examine the geometrical variability of PSS as a function of both the number of patients (nPat) and the number of stimulations per patient (nStim). The resulting findings were used to model a PSS stability boundary, offering a reference framework for identifying the combinations of nPat and nStim necessary to obtain geometrically stable PSS. Moreover, a second patient cohort was analyzed to assess the model’s generalizability to a different symptom. While direct evaluation of clinical impact is beyond the scope of this work, the study provides practical guidance on how cohort size and stimulation sampling influence the reliability of PSS-based analyses. This can inform both interpretation of results from previous studies and future study design.

## 2 Materials and methods

### 2.1 Patients and data–main cohort

The calculations were based on intra-operative stimulation test data from 36 patients diagnosed with Parkinson’s Disease (PD) who received DBS surgery between 2008 and 2016 at the Department of Neurosurgery of Clermont-Ferrand University Hospital, France. The patients were bilaterally implanted under local anesthesia targeting the subthalamic nucleus (STN), with the first implanted hemisphere being the left. Intra-operative stimulation tests were performed during surgery with a microelectrode (Alpha-Omega Engineering, Israel; frequency 130 Hz; pulse width 60 μs), exploring the STN area along two parallel trajectories and at positions spanning 14 mm with 1 mm step. Stimulation current amplitude, ranging from 0.2 to 3 mA, was increased in steps of 0.2 mA. Rigidity improvement was assessed by the neurologist as: “no improvement” (0%), “poor improvement” (25%), “fair improvement” (50%), “good improvement” (75%), “excellent improvement” (100%). In-between scores were also attributed if needed. The minimum current amplitudes that produced the greatest symptom improvement (motor thresholds) were recorded along with their corresponding improvement scores. In this study, data from the exploration of the first implanted hemisphere (left) were used. The dataset comprised a total of 527 stimulation tests, with the number of stimulations per patient ranging between 12 and 22. All patients signed a written informed consent (Ptolemee Electrophysiologie project: IRB 5921, CE-CIC-GREN-18-03) for the retrospective analysis of data.

### 2.2 PSS computation workflow

**FIGURE 1:**
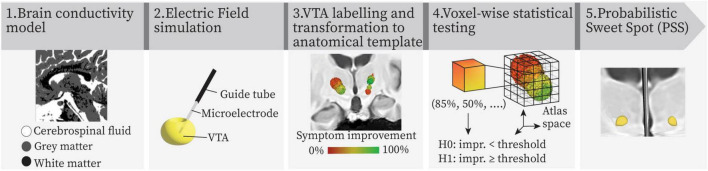
Summary of the main steps of the probabilistic mapping workflow. VTA: volume of tissue activated.

The main steps of the probabilistic mapping workflow are shown in Figure 1. Patient-specific brain conductivity models were generated with the software ELMA^1^ (Johansson et al., 2019) based on the patient’s pre-operative T1 MR images. ELMA classified the tissue into cerebrospinal fluid, white matter and gray matter. After extracting the electrodes’ coordinates in the patient’s reference space in 3D Slicer^2^ (Fedorov et al., 2012; Beninca et al., 2017), the spread of the current in the brain tissue was simulated in Comsol Multiphysics 5.5 (COMSOL AB, Sweden) for each stimulation setting (Åström et al., 2009). The simulation results were visualized as Electric Field (EF), and VTAs were obtained by thresholding at 0.2 V⋅mm^–1^. The threshold is the minimum value activating neuronal axons (Åstrom et al., 2015; Alonso et al., 2016). Details regarding the electrical conductivity models and simulation parameters can be found in our previous work (Hemm-Ode et al., 2016; Nordin et al., 2022). VTAs were labeled with the related symptom improvement score and transformed to a cohort-specific atlas (Vogel et al., 2020) for group analysis. Probabilistic Sweet Spots (PSS) were identified through voxel-wise statistical analysis using three methods. The first approach employed was a state-of-the-art technique widely adopted in probabilistic mapping studies: a one-sample, one-sided Wilcoxon signed-rank test combined with Benjamini–Hochberg False Discovery Rate (FDR) (Benjamini and Hochberg, 1995) voxel-wise correction (WFDR). The second statistical workflow introduced an alternative to the FDR by applying a voxel-wise nonparametric permutation-based correction to the Wilcoxon test (WPERM), with 200 permutations. Voxels exhibiting a corrected *p*-value < 0.05 were considered statistically significant and included in the resulting PSS volume, for both Wilcoxon-based methods. The third method involved a Bayesian one-sample *t*-test (BAYES), which has demonstrated promising performance in previous studies with respect to PSS stability and robustness to outliers (Bucciarelli et al., 2025b). Following a sensitivity analysis the chosen prior for BAYES was a normal distribution. A detailed description of the sensitivity analysis can be found in the Supplementary Figure 1 and Supplementary Table I. Samples from the posterior distribution of μwere obtained via Hamiltonian Monte Carlo implemented with Pyro/NUTS (No-U-Turn Sampler). Markov Chain Monte Carlo (MCMC) settings used in this study were 2000 num_samples and 1000 warmup_steps. Voxels were considered part of the PSS volume if they met a threshold of Bayes Factor ≥ 10 (Kruschke and Liddell, 2018). More detailed explanations of the statistical tests and corrections can be found in Bucciarelli et al. (2025a). A review in a previous study of our consortium (Stenmark Persson et al., 2022) indicated 50% as the median improvement for the symptom of rigidity. Based on this value, the null and alternative hypotheses were formulated as follows: H_0_: improvement < 50% and H_1_: improvement ≥ 50%. The significant voxels were further refined using a stimulation occurrence map (nMap) and a patient occurrence map (nPatMap). Specifically, voxels stimulated in fewer than 25% of the patients and with a stimulation frequency lower than 10% of the maximum stimulation occurrence (maximum nMap value) were discarded. Moreover, clusters smaller than 1 mm^3^ were excluded from the analysis, since their selection is likely to occur by chance due to random noise in the data (Forman et al., 1995; Woo et al., 2014).

### 2.3 Patients and stimulation sampling

**FIGURE 2:**
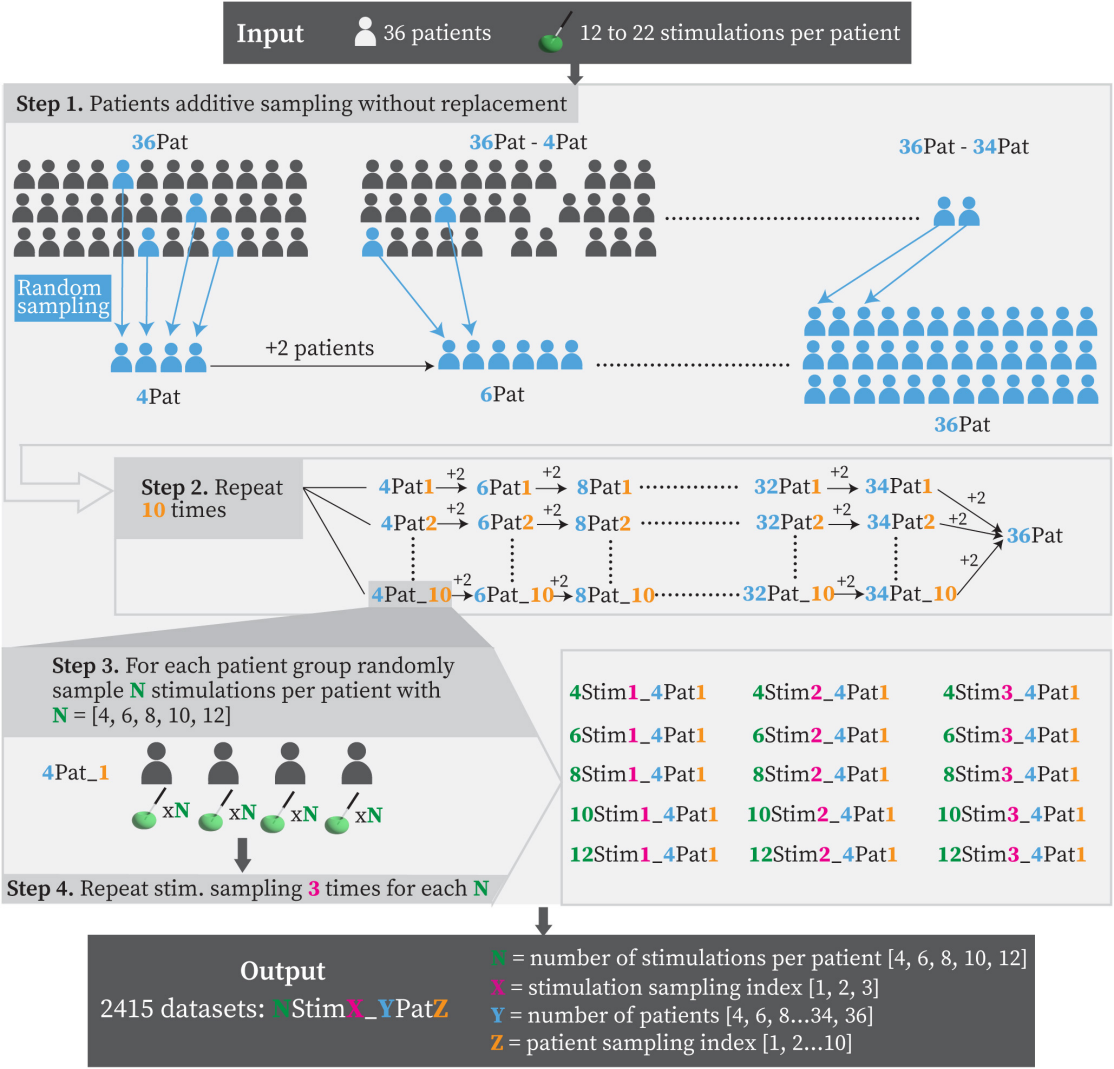
Graphical representation of patient and stimulation sampling strategy. Patient groups were generated starting from 4 randomly sampled patients and adding 2 of the remaining ones at each step. Each group’s sample size is reported in blue. Patient sampling was repeated 10 times (orange index). For each identified group of patients, subgroups were created by randomly sampling 4, 6, 8, 10 and 12 stimulations per patient (green number). The stimulation sampling was repeated 3 times (pink index), yielding a total of 2415 datasets.

The patient and stimulation sampling process is illustrated in Figure 2. To assess PSS variability across different sample sizes, patient subgroups were generated from the full cohort using an additive, sampling-without-replacement strategy. This sampling strategy was selected to reflect a realistic clinical scenario, in which a center incrementally collects data and updates its analyses as new patients become available. Each subgroup was initiated by randomly selecting 4 patients, followed by the iterative addition of 2 randomly chosen patients from the remaining pool at each subsequent step (step 1 in Figure 2). This procedure was repeated 10 times (patient sampling, step 2 in Figure 2), resulting in a total of 160 distinct datasets, in addition to the full cohort dataset. To further investigate the influence of the number of stimulations per patient on PSS computation, the previously generated 161 datasets were expanded by creating subsets with fixed stimulation counts. Specifically, for each dataset, new subsets were generated by randomly sampling 4, 6, 8, 10, and 12 stimulations per patient (step 3 in Figure 2). This process was repeated three times (stimulation sampling, step 4 in Figure 2) for each combination of patient sample size and stimulation count, resulting in a total of 2415 datasets used for PSS computation.

### 2.4 Comparison metrics and stability points

**FIGURE 3:**
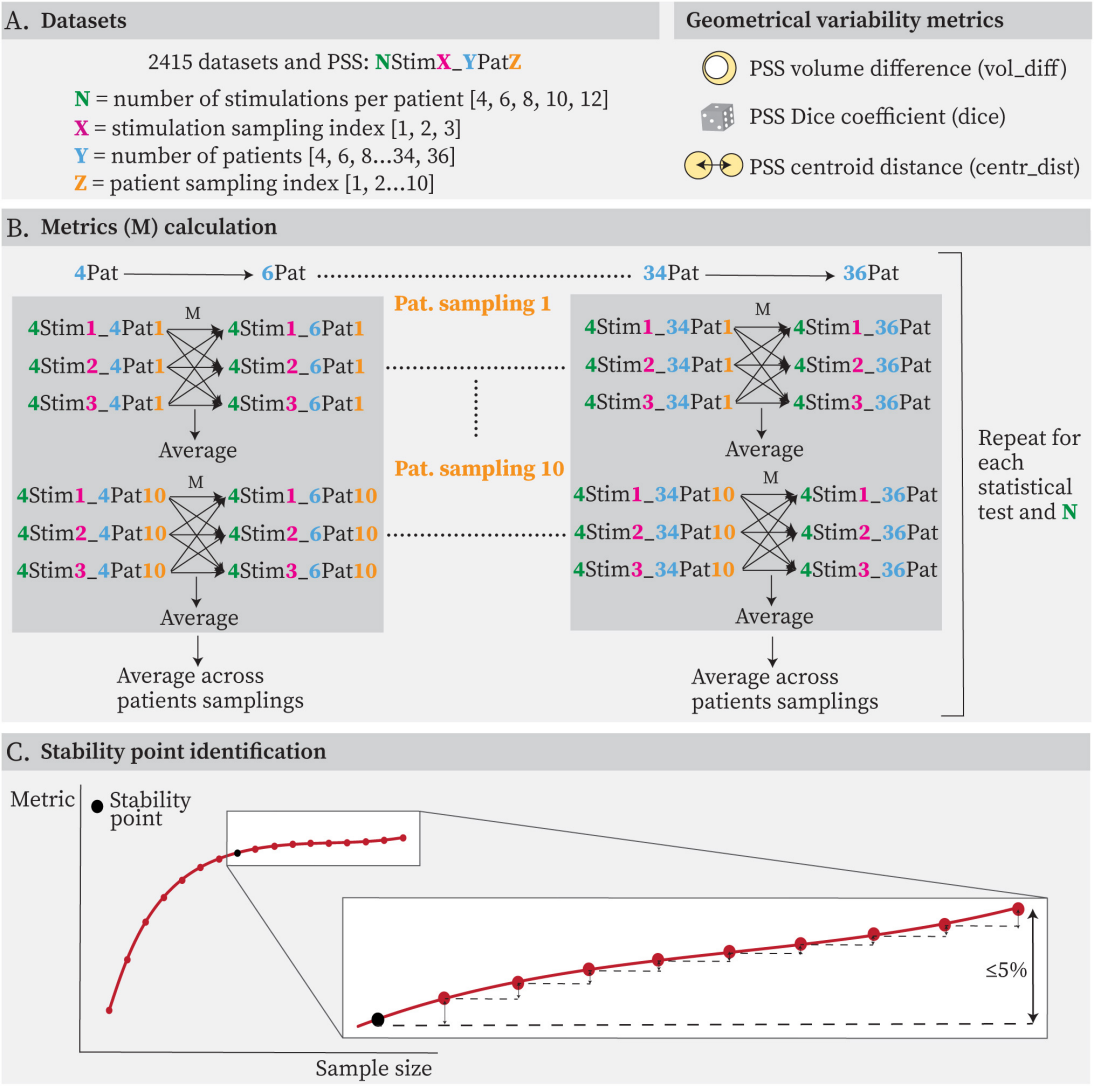
**(A)** Datasets generated from patient and stimulation samplings and metrics (M) chosen to evaluate the geometrical variability of the obtained Probabilistic Sweet Spots. **(B)** Graphical representation of the metrics calculation logic. For each statistical test and each number of stimulations per patient (N), the metric (M) was calculated between each possible combination of successive datasets (e.g., 4Pat and 6Pat, 6Pat and 8Pat) with different stimulation samplings. Results were then averaged between patient samplings (orange index). **(C)** Example of obtained curve for each metric, statistical test and stimulation count per patient (dark red) and criteria for identification of stability point (black). The stability point was defined as the earliest point after which the differences between this point and the curve’s final value remained below 5%.

Probabilistic Sweet Spots geometrical variability was assessed using a set of complementary metrics designed to capture different aspects of spatial deviation (Figure 3A). A stable PSS is expected to exhibit consistency in both location and volume. These were:

- Volume difference normalized by the largest volume in the pair [%] (vol_diff)
- Dice coefficient [%] (dice)
- Centroid distance normalized by the largest diameter in the pair [%] (centr_dist)

Absolute volumes and absolute centroid distances were also calculated. For each statistical test and stimulation count per patient, variability metrics were computed between successive dataset sizes, as illustrated in Figure 3B. Specifically, comparisons were made between datasets with 4 and 6 patients, 6 and 8 patients, and so forth. Each stimulation count was sampled three times. Metrics were therefore computed across all possible pairwise combinations of successive datasets and averaged. These values were then further averaged across the 10 patient samplings, resulting in a curve for each combination of statistical test and stimulation count per patient. A curve was deemed stable when the difference between the identified stability point and the final value remained under 5% (Figure 3C).

### 2.5 Number of patients–number of stimulations trade-off modeling

**FIGURE 4:**
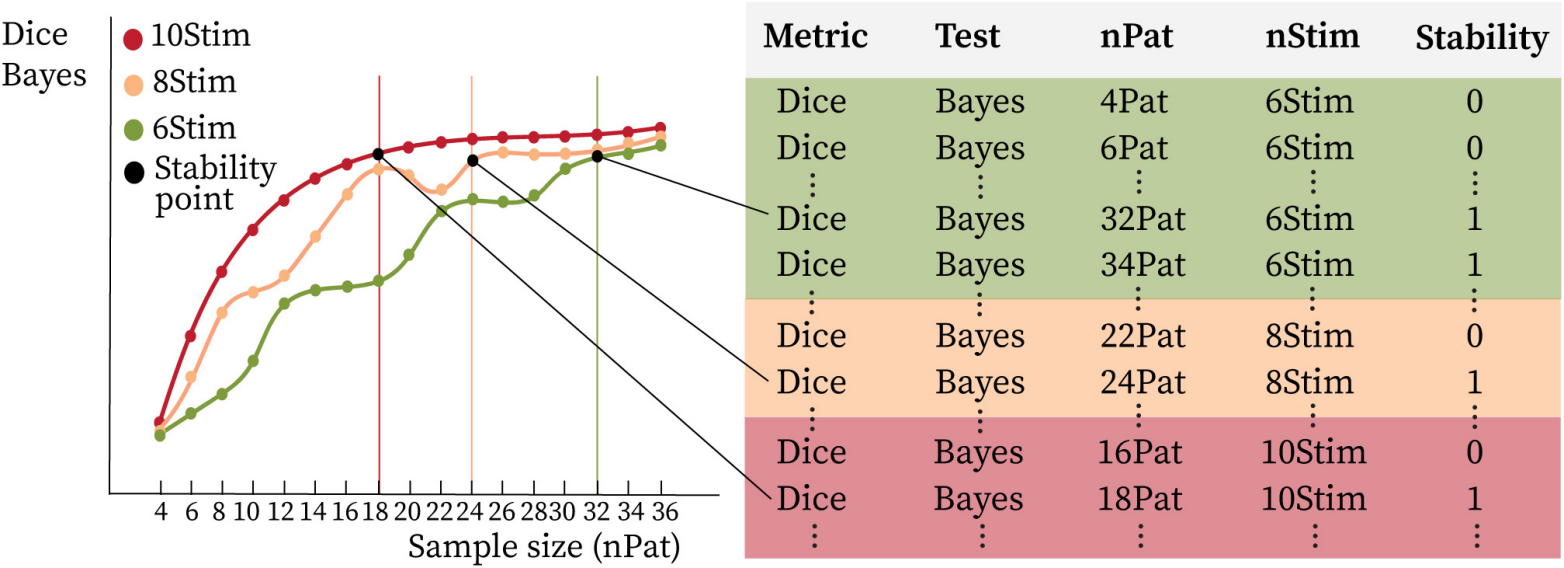
Graphical representation of the generation of stability information for the different stimulation sampling sizes. On the left, example curves obtained for each metric and statistical test are shown. Each combination of metric, statistical test, number of patients (nPat) and number of stimulations per patient (nStim) corresponds to a record in the dataset and has an associated binary stability label (on the right). Sample sizes larger than the one corresponding to the stability point (black dot on the curve) are labeled as 1, while smaller ones are labeled as 0. Considering the example curve in the figure, for 6Stim (green), nPat < 32 have stability 0, while nPat ≥ 32 have stability 1. Stability values exist for each data point (dots on the curves on the left), the table shown in the figure reports only some example values.

The stability outcomes derived from each statistical test, geometric metric, and stimulation count were utilized to model the trade-off between the number of patients and the number of stimulations per patient. To achieve this, stability was treated as a binary outcome variable, assigned to each unique combination of patient count and per-patient stimulation count. Specifically, each pair (number of patients, stimulation count) was labeled as either 0 (stability not achieved) or 1 (stability achieved), thereby forming a binary stability dataset. Additionally, the total number of stimulations, computed as the product of the number of patients and the number of stimulations per patient, was included as a summary variable, capturing the combined contribution of both sample size and stimulation count. The dataset generation is graphically illustrated in Figure 4. A binary classification model based on the Bayesian Logistic Regression algorithm was trained on the constructed dataset. The model performed inference using MCMC sampling. For each patient sample size, we computed the minimum number of stimulations per patient required to reach a probability of stability ≥ 0.5 for each posterior sample. This procedure generated a distribution of stimulation thresholds, from which we inspected the mean and the 95% credible interval, representing the uncertainty of the estimated boundary due to posterior variability. The total number of stimulations was used as the input feature and the binary stability label as the target variable. The model was independently trained for each combination of statistical test (WFDR, WPERM and BAYES) and geometric metric. Additionally, the model was also trained on aggregated data from all metrics, while maintaining separate models for each statistical test. The trained models were subsequently employed to predict the minimum number of stimulations per patient required to achieve PSS stability across a range of sample sizes, varying from 4 to 100 patients. Model performance was evaluated using a 70%/30% train–test split, and accuracy and F1 score were used as evaluation metrics to assess predictive validity.

### 2.6 Comparison with a second cohort

A second cohort, comprising 61 Essential Tremor (ET) patients, was used to investigate the model extrapolation outside the original cohort size and generalizability to another symptom and DBS phase (chronic screening data). The patients had received DBS in the caudal zona incerta (lead 3387 or 3389, Medtronic, Minneapolis, MN, USA) at Norrlands University Hospital in Umeå, Sweden. For each patient and DBS contact, the lowest voltage that generated the best improvement was registered together with the improvement in Essential Tremor Rating Scale (ETRS), items 5/6 and 11–14. This resulted in 4 stimulations for each patient, i.e., a total of 244 stimulations. For more details on the dataset, see previous publications (Nordin et al., 2022, 2023). All patients signed a written informed consent, and the study was approved by the local Ethics Committee at Umeå University (Dnr. 01-123M, 2017/122-31). All stimulations were simulated using the same method as previously described with the addition of the peri-electrode space simulation (Nordin et al., 2022). This was then followed by computation of PSS with WFDR, WPERM and BAYES. Given the different evaluated symptom (tremor), similarly to the first cohort, the improvement threshold to test against was set at 75%, based on the findings reported by Stenmark Persson et al. (2022). The stability points identified by each metric for the ET cohort were compared with the ones found for the PD cohort at a stimulation count of 4. Additionally, an average metric was computed by combining volume differences (vol_diff), Dice coefficients (dice), and centroid distances (centr_dist): m_comb = mean (vol_diff, dice, centr_dist). This average metric served to evaluate whether the stability point aligned with the prediction derived from the Bayesian Logistic Regression model trained on all metrics from the PD cohort. A greater discrepancy between these values indicates lower generalizability of the model to different clinical indications and phases of DBS.

## 3 Results

### 3.1 Stability points for PD cohort

**FIGURE 5:**
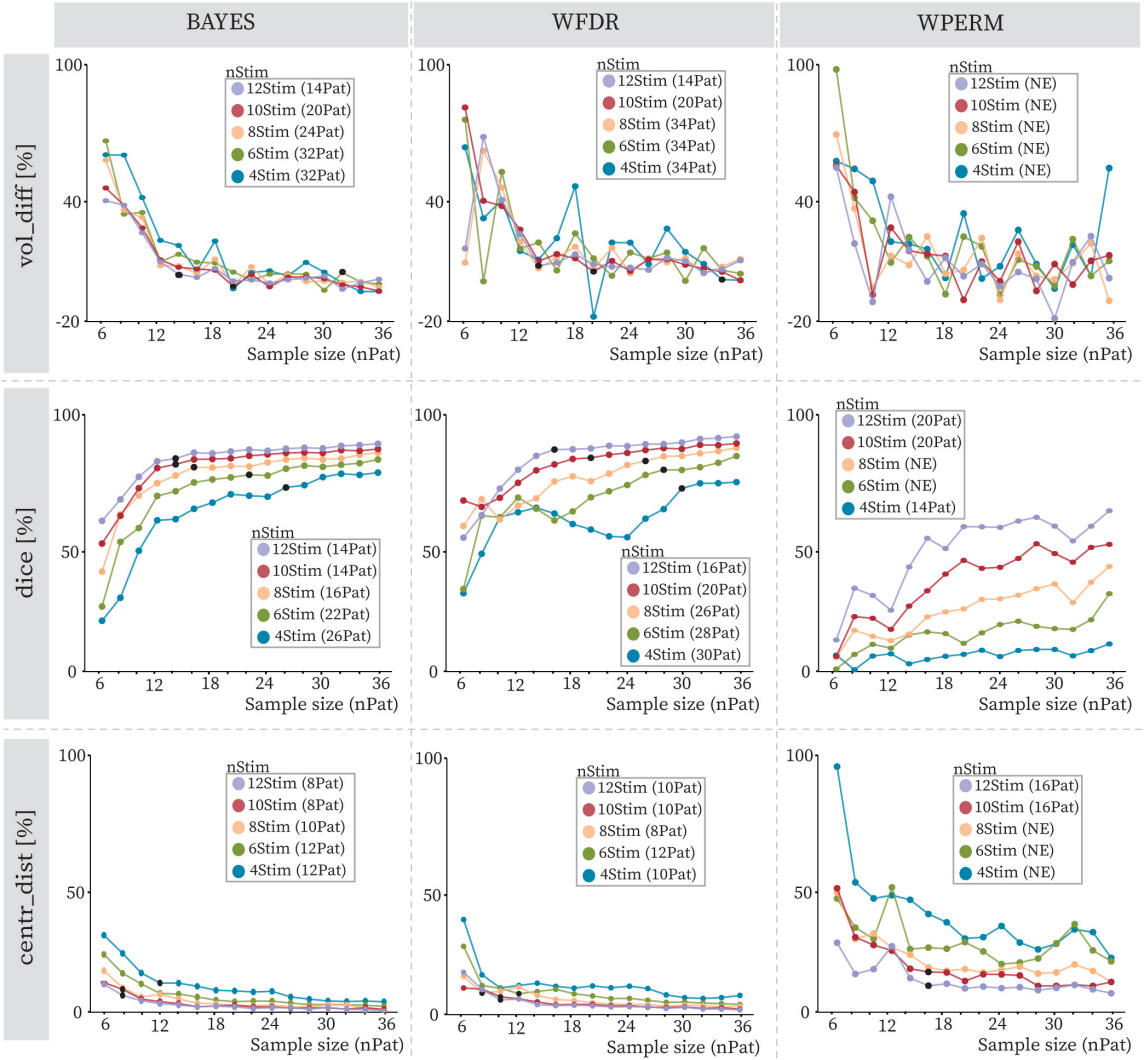
Volume differences (vol_diff), Dice coefficients (dice) and centroid distances (centr_dist) with increasing sample size (nPat) and different stimulation counts (nStim) for BAYES (left), WFDR (middle) and WPERM (right) for the PD cohort. The dots represent average values across patient samplings, and the line color refers to the stimulation count as reported in the legend (4Stim: blue, 6Stim: green, 8Stim: pink, 10Stim: red, 12Stim: violet). The value in parentheses near the stimulation count in the legend indicates the number of patients at which the stability point (black dot), if existing, was identified. NE (not existing) indicates that no stability point was found.

Figure 5 illustrates the behavior of the computed geometrical variability metrics as a function of increasing sample size for the PD cohort. A separate curve was generated for each combination of statistical test, stimulation count per patient, and metric. The standard deviations for each curve are shown in Supplementary Figure 2. The general trend for all curves was a decrease in the required patients with increasing number of stimulations per patient (e.g. 26 patients for 4Stim, 22 patients for 6Stim, 16 patients for 8Stim and 14 patients for both 10Stim and 12Stim for Dice coefficient with BAYES). Across all statistical tests, the centroid distance metric demonstrated stability points at smaller sample sizes, followed by the Dice coefficient and volume difference. For example, with BAYES and 10Stim stability was reached at 8 patients, 14 patients and 20 patients for centr_dist, dice and vol_diff, respectively. The BAYES approach exhibited smoother curve stabilization across all metrics, in contrast to WFDR and WPERM, which showed greater fluctuations. Among them, WPERM yielded the lowest Dice coefficients (lower than 55% and below 10% for 4Stim) and the highest volume differences and centroid distances. Additionally, BAYES demonstrated the most consistent behavior across curves generated with varying numbers of stimulation trials. Examples of obtained PSS in the anatomical atlas are shown in Supplementary Figure 3, while absolute volumes [mm^3^] and absolute centroid distances [mm] are shown in Supplementary Figures 4, 5, respectively. WPERM provided considerably smaller PSS volumes with respect to BAYES and WFDR.

### 3.2 Stability points for the ET cohort

**FIGURE 6:**
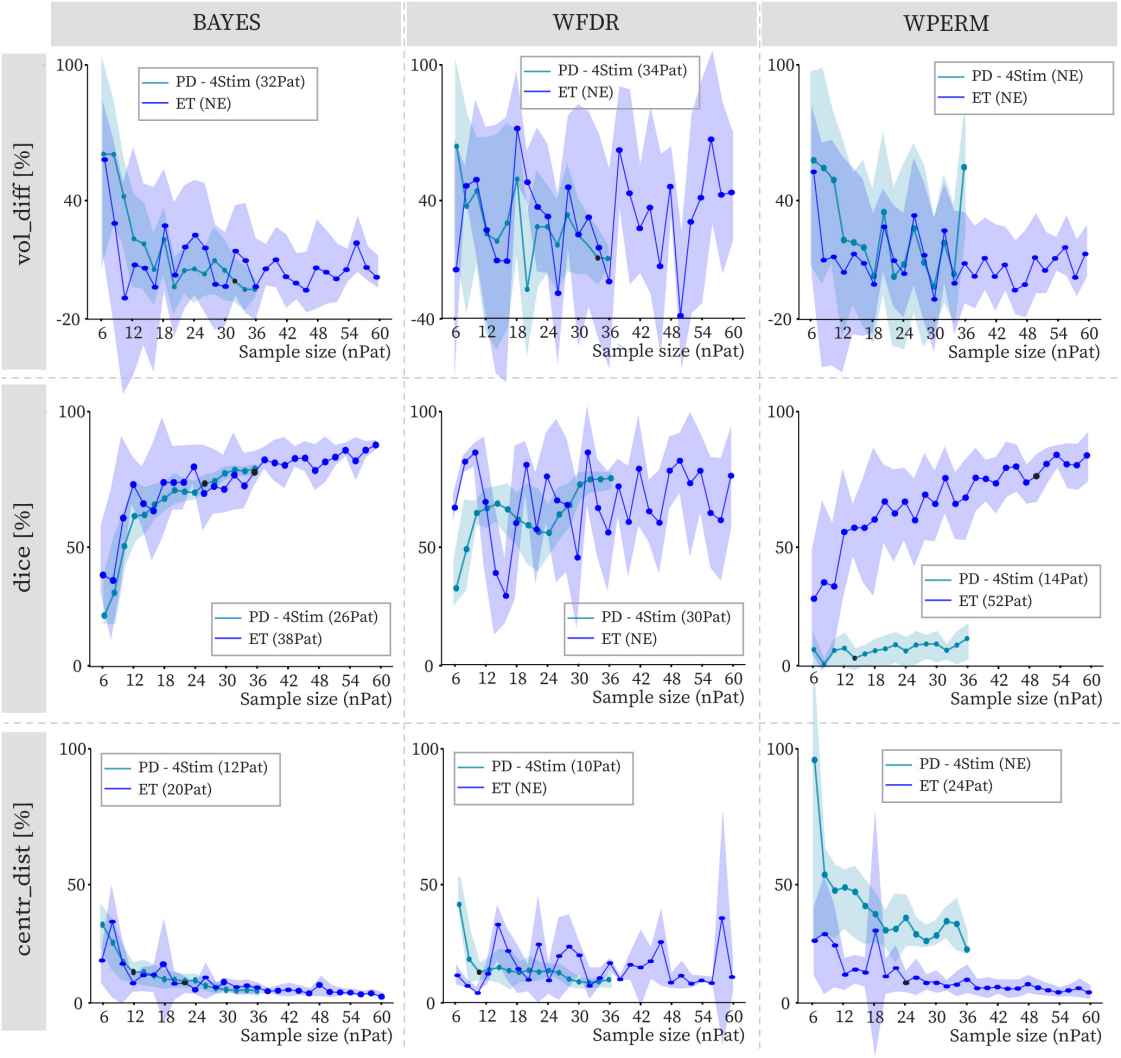
Volume differences (vol_diff), Dice coefficients (dice), and centroid distance (centr_dist) variation with increasing sample size (nPat) with stimulation count of 4Stim for BAYES (left), WFDR (middle) and WPERM (right). The dots represent average values across patient samplings. The line referring to the ET cohort is in dark blue, while the lighter one is for the PD cohort with 4Stim for comparison. The shaded area around the line represents the standard deviation. The value in parentheses near the stimulation count in the legend indicates the number of patients at which the stability point (black dot), if existing, was identified. NE (not existing) indicates that no stability point was found.

Figure 6 illustrates the evolution of the geometrical variability metrics for 4 stimulations in relation to increasing sample size within the ET and PD cohorts. For ET, none of the statistical methods achieved stability for volume differences, and WFDR failed to reach stability across all metrics. In contrast, BAYES reached stability at 38 patients for the Dice coefficient and at 20 patients for centroid distance. WPERM stabilized at 52 patients for Dice and 24 for centroid distance. Stability was achieved less frequently in the ET cohort compared to the PD cohort. In only one combination, centroid distance for WPERM, did the ET cohort reach stability where the PD cohort did not. Moreover, when stability points were present in both groups, they occurred at higher patient numbers in the ET cohort than in the PD cohort. The largest discrepancy between the cohorts was seen for the WPERM, especially for the dice and centroid distance metrics. Absolute volumes and centroid distances are shown in Supplementary Figures 6, 7. In this cohort, WFDR consistently produced smaller PSS volumes compared to both BAYES and WPERM.

### 3.3 Number of patients–number of stimulations trade-off

**FIGURE 7:**
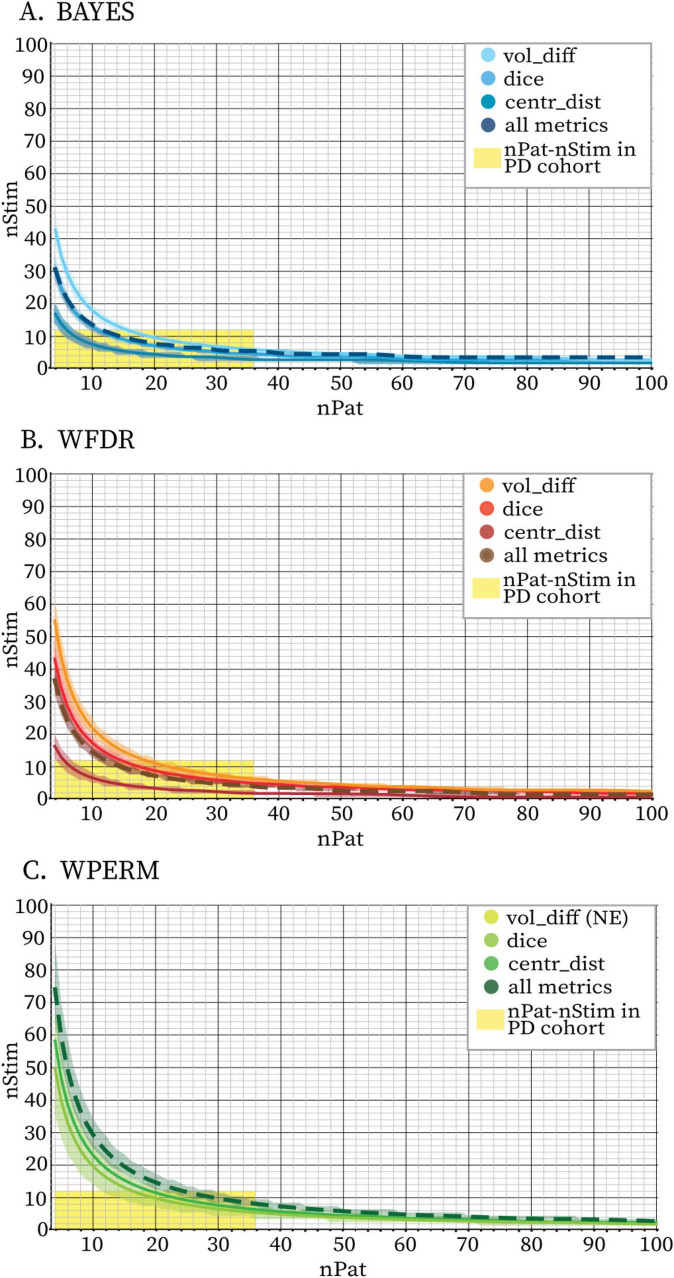
Stability boundaries predicted by the Bayesian Logistic Regression model fit on volume differences only (vol_diff, full line), Dice coefficients only (dice, full line), centroids distances only (centr_dist, full line) and all three metrics together (all metrics, dashed line) for BAYES (blue color scale) **(A)**, WFDR (red color scale) **(B)** and WPERM (green color scale) **(C)** for the PD cohort. The stability threshold was 5%. The lines represent the mean sample sizes estimated by the model for PSS stability and the shaded area around the line represents the 95% credible interval. Combinations of nPat-nStim falling above the stability boundary are likely to yield PSS with stable volumes and locations. The area highlighted in yellow indicates the combinations of nPat-nStim available in the dataset (maximum 36 patients and 12 stimulations per patient) and which were used to fit the model. The model was not able to identify a stability boundary for WPERM based on the vol_diff metric (NE = not existing). For all three statistical tests, the curve based on the aggregated metrics closely aligned with the Dice-based curve.

Figure 7 presents the predictions of the Bayesian Logistic Regression model trained on the stability dataset extracted from the Clermont-Ferrand PD cohort data. The figure illustrates the minimum number of stimulations per patient required to achieve stable PSS as a function of patient sample size. The model captured a non-linear, exponentially decaying relationship between the number of patients (nPat) and the required stimulation count (nStim) across all the statistical methods and evaluated metrics. Due to variations in the sample sizes at which stability was achieved for different metrics, the predicted stability boundaries also differed accordingly. These differences were most pronounced at smaller sample sizes and gradually converged at larger ones, where the curves tended to plateau. When trained on aggregated data combining vol_diff, dice, and centr_dist metrics, the model produced a curve that consistently lay between those derived from individual metrics. Among the metrics, centr_dist required the fewest stimulations to achieve stability, while vol_diff required the most, with dice falling in between. For WPERM, the model was not able to predict a stability boundary when basing only on vol_diff, since volume differences did not reach stability for any stimulation count. The model exhibited higher confidence in predicting the stability boundary for the BAYES approach, as reflected by narrower 95% credible intervals. In contrast, WPERM displayed the widest credible intervals, while WFDR showed intermediate uncertainty. Additionally, the total number of stimulations predicted for achieving stability differed between the three statistical tests when considering all three metrics together: BAYES required fewer total stimulations (approximately 125–175) compared to WFDR (approximately 150–200) and WPERM (250–300). Overall, the model demonstrated strong predictive performance across the evaluated metrics and statistical tests (Supplementary Table II). Accuracy and F1 scores were consistently high, particularly for the BAYES and WFDR approaches. Performance was slightly lower for WPERM in some metrics, notably dice and all the metrics combined, reflecting increased variability in these predictions.

**FIGURE 8:**
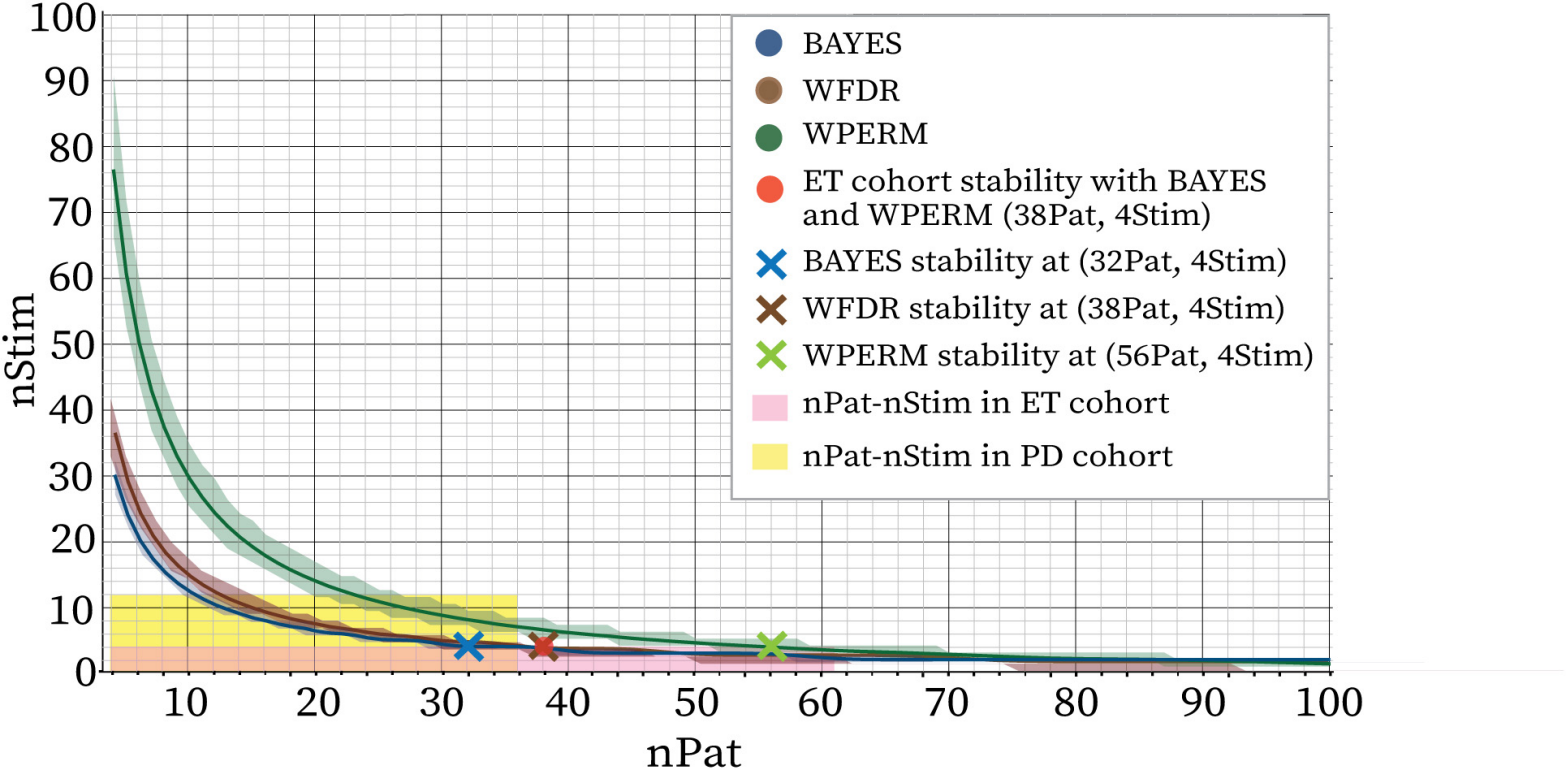
Stability boundaries predicted by Bayesian Logistic Regression model fit on all the metrics together for PD cohort for BAYES (blue), WFDR (brown) and WPERM (green). The stability threshold was 5%. The lines represent the mean sample sizes estimated by the model for PSS stability and the shaded area around the line represents the 95% credible interval. The area highlighted in yellow indicates the combinations of nPat-nStim available in the PD dataset (maximum patient number of 36 and maximum stimulations per patient 12), while the pink area indicates the nPat-nStim combinations available in the ET dataset (maximum patient number of 61 and maximum stimulations per patient 4). The red dot indicates the nPat-nStim combination at which stability was reached for the average metric (m_comb) calculated for the ET dataset for BAYES and WPERM (nPat: 38, nStim: 4). WFDR did not provide a stability point on this dataset. The cross symbols indicate the estimated minimum nPat corresponding to 4Stim for BAYES (blue, 32Pat), WFDR (brown, 38Pat) and WPERM (green, 56Pat).

Figure 8 presents the combined average curves for BAYES, WFDR, and WPERM from Figure 7, plotted together to facilitate visual comparison. The red dot indicates the specific nStim–nPat combinations at which the combined metric (m_comb) for the ET cohort reached stability (Supplementary Figure 8). No stability point was identified for WFDR, whereas both BAYES and WPERM achieved stability at 38 patients.

## 4 Discussion

The dependence of PSS on input data is a frequently overlooked aspect when evaluating the reliability and generalizability of results. Building upon previous work (Nordin et al., 2023; Bucciarelli et al., 2025b), we have investigated the geometrical variability of PSS as a function of both patient sample size and number of stimulations per patient. Furthermore, a stability boundary was modeled to provide guidance on the combinations of nPat and nStim that yield geometrically stable PSS. Such a reference framework can serve as a valuable tool in informing the design of data acquisition protocols in future studies. Combinations of nPat and nStim falling below the identified stability boundary may result in PSS that are more strongly influenced by individual electrode positions. Multiple patient and stimulation samplings enabled us to account for inter-patient variability and thereby enhanced the robustness of the findings. To assess generalizability, the analysis was repeated on a separate patient cohort undergoing DBS for a different primary symptom (tremor instead of rigidity) and evaluated during another phase of the procedure (post-operative screening rather than intra-operative testing).

### 4.1 Impact of sample size and methods comparison

As previously reported in Bucciarelli et al. (2025b), the sample size required for PSS stabilization varies depending on the selected metric and statistical test. Notably, centroid distances reached stability at smaller sample sizes compared to volume-based metrics such as the Dice coefficient and volume differences. This suggests that the spatial location of the PSS becomes consistent with relatively few patients, whereas the inclusion of additional VTAs primarily affects its volumetric extent. Aligning with previous observations (Bucciarelli et al., 2025b), BAYES generally achieved PSS stability at smaller sample sizes compared to WFDR and WPERM, thereby suggesting greater consistency in its performance. The observed trends – namely, earlier stability for centroid distances and for the BAYES method – were also confirmed in the analysis of the ET cohort. However, the minimum sample sizes required to achieve stability varied across metrics and methods when compared to those identified in the 4Stim-PD cohort. These differences were particularly pronounced for the WFDR and WPERM methods. WFDR demonstrated better convergence in the PD cohort but exhibited substantial oscillations in the ET cohort, whereas WPERM showed the opposite pattern. The fluctuations may be attributed to the identification of very small PSS volumes by WFDR in the ET cohort and by WPERM in the PD cohort. The very different performances of WFDR and WPERM in the two cohorts could reflect their sensitivity to the spatial patterns in the EF distributions or the distribution of improvement scores. In particular, the PD cohort exhibited a more uniform improvement score distribution with a mean around 50, whereas the ET cohort was skewed toward higher values with a mean around 70. The distribution of improvement scores may therefore represent an additional factor to consider, and it would be of interest to investigate how it influences the results. Conversely, the BAYES method showed consistent performance across both cohorts, indicating that it may generalize across different symptom profiles. However, given the differences in cohort size, symptom type, and improvement score distributions, further analyses are needed to confirm this potential. In general, stability was reached less frequently or at a higher nPat in the ET cohort with respect to the PD cohort. A different minimum sample size was reported by Nordin et al. (2023), who analyzed the same ET cohort used for this study. The discrepancy may be attributed to several factors, such as the used statistical test (*t*-test + permutations vs. Wilcoxon test + permutations), chosen improvement threshold (70% vs. 75%), evaluation metrics, or the sampling strategy used to generate patient subgroups (additive sampling without replacement vs. sampling with replacement). In particular, the choice of a sampling-with-replacement strategy, which allows individual patients to appear multiple times in a sampled subgroup, is inherently expected to increase variability and shift the estimated stability point to larger sample sizes.

### 4.2 nPat-nStim trade-off modeling

When evaluating the combined effect of patient sample size and stimulation count on PSS stability, analysis of individual metrics suggested a non-linear, inverse relationship between the two factors (e.g. larger sample sizes appeared to require fewer stimulations per patient to achieve stability, and vice versa). This trend was further supported by the stability modeling. In the model, the total number of stimulations was selected as the independent variable, as it effectively captures the joint contribution of both nPat and nStim. The identified trade-off between patient sample size (nPat) and stimulation count (nStim) exhibited an exponentially decaying trend, consistent across all geometrical metrics and statistical tests. The primary differences among the metrics were observed in the initial values of the curve, specifically, the predicted nStim required for stability at small sample sizes (e.g., nPat = 4). The model estimated lower stimulation counts necessary for achieving PSS stability when centroid distances were used, compared to the Dice coefficient or volume differences. This aligns with the data, which showed that the spatial location of the PSS stabilizes at smaller sample sizes than its volumetric properties. When aggregating results from all three metrics, the model effectively performed a form of majority voting, resulting in an average curve that lay between those derived from the individual metrics. Based on this aggregated curve, BAYES was found to require less data than WFDR or WPERM to produce geometrically stable PSS, highlighting its suitability for small patient cohorts. Based on the model, with four stimulations per patient, stability was observed at 32 patients for BAYES, which is within the available experimental data. Model projections beyond this sample suggest stability at 38 patients for WFDR, and 56 patients for WPERM; however, these extrapolated values should be interpreted with caution, as non-linear model predictions beyond the observed sample may be unreliable. Keeping this possible unreliability in mind, we compared the model-derived stability points with the findings from the ET cohort, which do not fully align, although the deviation is not far from the prediction with BAYES. For the ET cohort stability–based on the combined metrics–was observed at 38 patients for both BAYES and WPERM, and stability was not reached for WFDR. The substantial discrepancies observed for WFDR and WPERM suggest limited generalizability of these methods across different cohorts or symptoms. In contrast, the relatively small difference in stability points for BAYES between the PD and ET cohorts indicates a greater potential for generalizing the method cross-cohort - both across different clinical indications (e.g., Parkinson’s Disease vs. Essential Tremor) and across different data acquisition contexts (intra-operative vs. post-operative) - with minimal adjustments. Further investigation is required to better understand and validate this relationship.

### 4.3 Strengths and limitations

This study was enabled by the richness of the PD dataset, particularly the large number of stimulation tests available per patient. In contrast, most DBS datasets typically include only a limited number of tests per individual or focus solely on the optimal stimulation settings. The moderate size of the patient cohort was a limitation, as it constrained the amount of data available for model training. As a result, the model’s predictions for regions beyond 36 patients or 12 stimulations per patient could not be directly validated against experimental observations. While the model offers a valuable reference for guiding data acquisition, its extrapolations in these areas may be less accurate and would benefit from integration of larger patient cohorts. According to the model, stability can also be achieved with smaller patient samples if stimulation counts are high; however, users should be aware that results derived from very few patients (e.g., 2 or 4) are unlikely to be robust or generalizable. Another strength of the study was the inclusion of a second patient cohort, providing an initial indication of the translatability of the findings to a different symptom or condition and to a different type of stimulation testing. Although limited to four stimulations per patient, this dataset still provided valuable insight into the broader applicability of the results. Another source of variability in our analysis arises from the choice of voxel inclusion thresholds (≥25% of patients and ≥10% of the maximum stimulation occurrence) and the selection of the improvement threshold in the PSS results. While percentage-based voxel thresholds facilitate normalization across studies, they inherently depend on cohort size, and using absolute voxel counts could be an alternative. At the same time, relaxing the thresholds might increase the number of included voxels at the potential cost of reduced volumetric stability^3^. Similarly, although the improvement threshold was chosen based on clinically relevant values from existing literature, it nonetheless influences the outcomes. Finally, a curve was deemed to have reached stability when the difference between the identified stability point and its final value remained below 5%. Although this threshold reflects a common engineering convention, its selection is arbitrary. Consequently, tightening or relaxing this criterion would be expected to increase or decrease the minimum sample size required to achieve PSS stability.

## 5 Conclusion

This study examined the geometrical variability of PSS in relation to both the number of patients and the number of stimulations per patient. The findings were used to develop a model defining a stability boundary, which identifies the minimum nPat–nStim combinations required to achieve stable PSS–i.e., maps whose location and extent are less affected by the electrode positions of individual patients. This stability boundary offers a valuable reference for assessing the generalizability of probabilistic mapping results from cohorts with specific compositions. Furthermore, it can guide the design of stimulation screening protocols intended for subsequent use in probabilistic mapping analyses. Among the methods, the Bayesian *t*-test achieved the best stability with the smallest number of samples and exhibited the most consistent performance across both cohorts. This supports its suitability for probabilistic mapping in studies with limited patient numbers and its potential applicability across different symptom profiles. In contrast, notable discrepancies were observed in the behavior of the Wilcoxon-based methods between the cohorts, particularly in relation to symptom type and testing phase (intra-operative vs. post-operative), highlighting the sensitivity to cohort distribution and the need for caution when employing these methods.

## Data Availability

The datasets presented in this article are not readily available because of the requirement of participant’s approval for data sharing as requested by the ethics. Requests to access the datasets should be directed to the corresponding author.

## Footnotes

1
https://liu.se/en/article/ne-downloads

2
https://www.slicer.org/

3 Bucciarelli, V., et al. (Submitted). *How statistical methods, hemispheric data and masking approaches shape probabilistic sweet spots in deep brain stimulation. Manuscr. Submitt. Publ*.
