# Supplementary material for: Towards robust probabilistic maps in Deep Brain Stimulation: exploring the impact of patient number, stimulation counts, and statistical approaches
Source: Front Comput Neurosci. 2026 Jan 21;19:1699192. doi: 10.3389/fncom.2025.1699192 (PMC12868180; doi:10.3389/fncom.2025.1699192)
Supplement: Supplementary file 1 [file Supplementary_file_1.docx]

Supplementary Material

# Bayesian sensitivity analysis

A sensitivity analysis was performed to inform the choice of the prior distribution for the Bayesian t-test calculating the metrics reported in (Depaoli et al. 2020) on a sample of voxels for normal, T-student and Cauchy priors. The metrics are:

- Model convergence evaluation by considering the R-hat and effective sample size (ESS) values. In particular, R-hat<1.01 and ESS>1000 indicate model convergence and enough precision in the chain.
- Posterior distribution visual inspection: The posterior distribution is the probability distribution that represents updated beliefs about an unknown parameter after observing new data. If the posterior distributions are very similar it means that the posterior estimate is robust to different priors (and therefore the different priors would yield approximately the same results).
- Percentage deviation in the average posterior estimate computation: a low percentage deviation means high robustness to prior. A posterior estimate is a summary value (mean in this case) derived from the posterior distribution. The percentage deviation estimates how much the average posterior estimate differs from the true value.
- Bayes Factor comparison and consequent voxel classification.

**Table SI** and **Figure S1** report example results obtained on some of the voxels involved in the sensitivity analysis for both the PD and ET cohorts. The data showed robustness to the prior choice (e.g. the choice of different priors did not influence the final results), so a normal prior was chosen for the sweet spot calculation.

Table SI. Sensitivity analysis numerical metrics calculated for some example voxels and three prior distributions.

| Voxel | Prior distribution | ESS | R-hat | % dev. | BF |
| --- | --- | --- | --- | --- | --- |
| Voxel 1 - PD | Normal | 1694 | 1.0 | -0.54 | 21.72 |
| Voxel 1 - PD | Cauchy | 1646 | 1.0 | -0.53 | 24.0 |
| Voxel 1 - PD | T-student | 1592 | 1.0 | -0.62 | 22.95 |
| Voxel 2 - PD | Normal | 1497 | 1.0 | -0.18 | 15.67 |
| Voxel 2 - PD | Cauchy | 1533 | 1.0 | -0.43 | 14.03 |
| Voxel 2 - PD | T-student | 1837 | 1.0 | -0.3 | 12.84 |
| Voxel 3 - ET | Normal | 1609 | 1.0 | -0.042 | 1.35 |
| Voxel 3 - PD | Cauchy | 1706 | 1.0 | 0.01 | 1.39 |
| Voxel 3 - PD | T-student | 1905 | 1.0 | -0.03 | 1.41 |
| Voxel 4 - PD | Normal | 1553 | 1.0 | 0.04 | 2.99 |
| Voxel 4 - PD | Cauchy | 1812 | 1.0 | -0.004 | 2.88 |
| Voxel 4 - PD | T-student | 1816 | 1.0 | -0.08 | 2.86 |


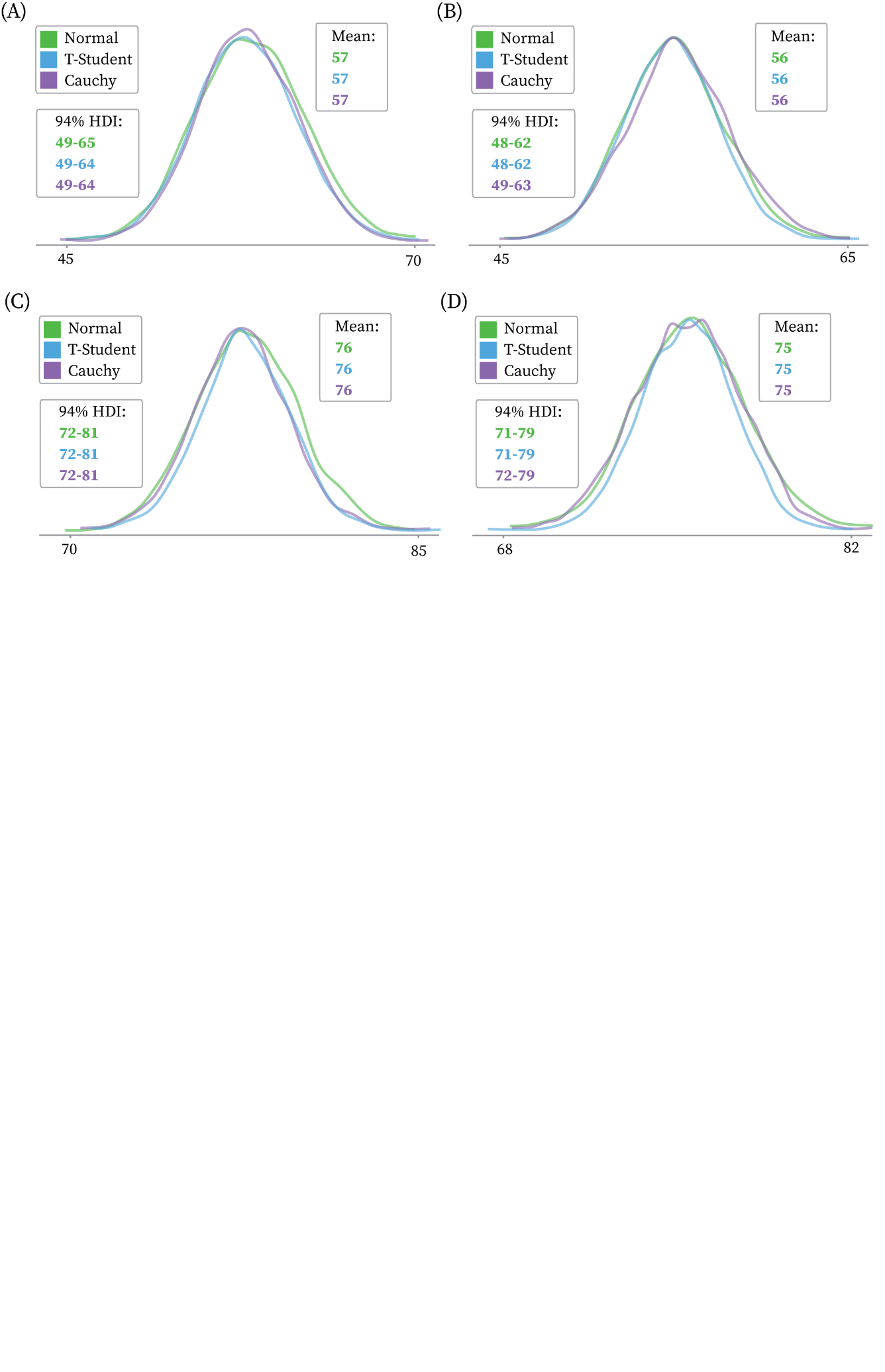


**Supplementary Figure S1.** Posterior distributions plots for the normal, T-student and Cauchy prior for example voxel 1 (A), voxel 2 (B), voxel 3 (C) and voxel 4 (D). The mean values and 94% highest posterior density intervals (HDI) values are reported in the boxes.

# Standard deviations for stability metrics


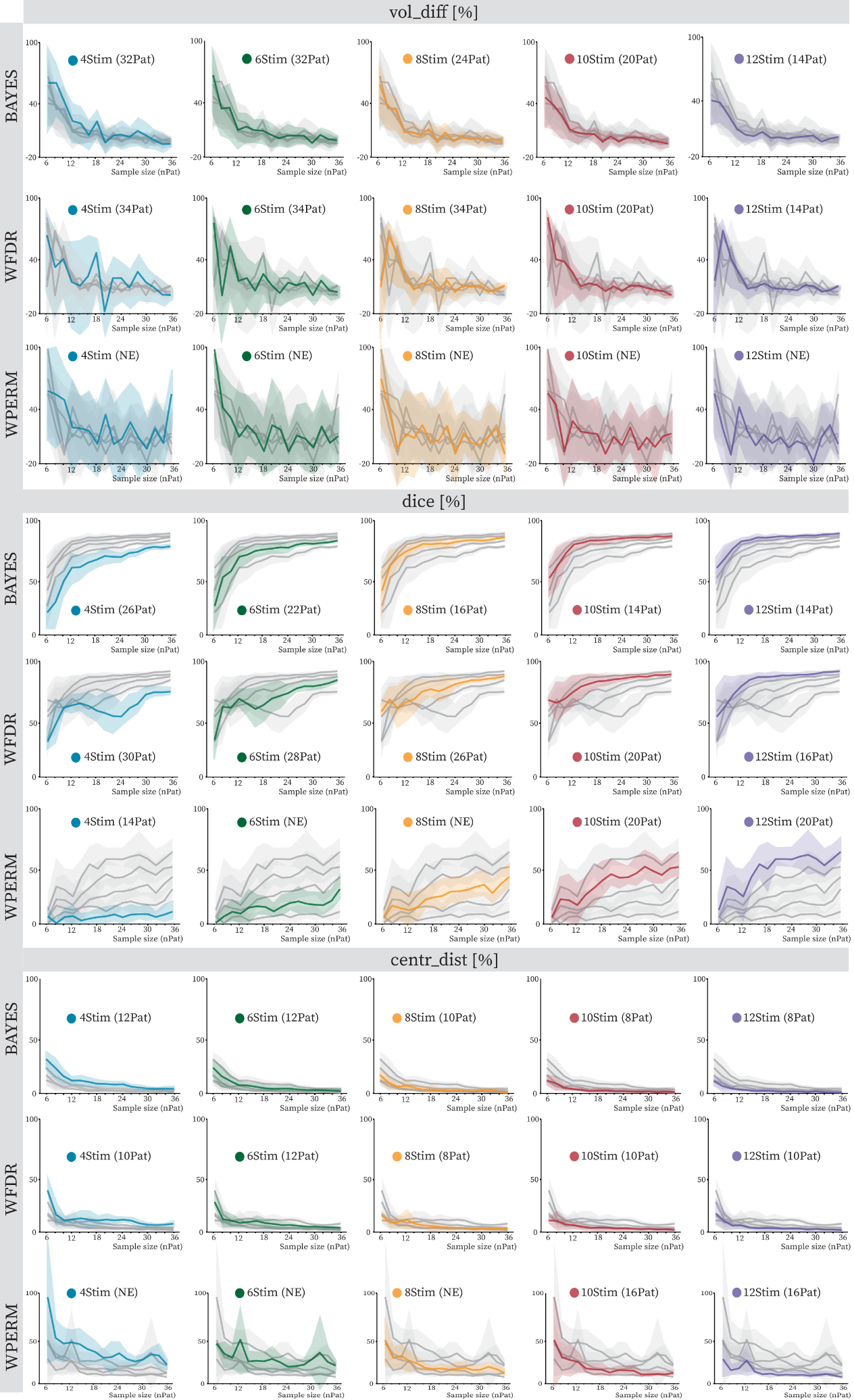


**Supplementary Figure S2**. *Volume differences (vol_diff), Dice coefficients (dice), and centroid distances (centr_dist) with increasing sample size (nPat) and different stimulation counts (nStim) for BAYES, WFDR and WPERM for the PD cohort. The lines represent average values across samplings and blurred areas represent the standard deviations. The color refers to the stimulation count as reported in the legend (4Stim: blue, 6Stim: green, 8Stim: pink, 10Stim: red, 12Stim: violet). The value in brackets near the stimulation count in the legend indicates the number of patients at which the stability point, if existing, was identified. NE (not existing) indicates that no stability point was found. For better readability each graph highlights one curve for each stimulation count, with the other stimulation counts shaded in grey.*

# Examples of PSS


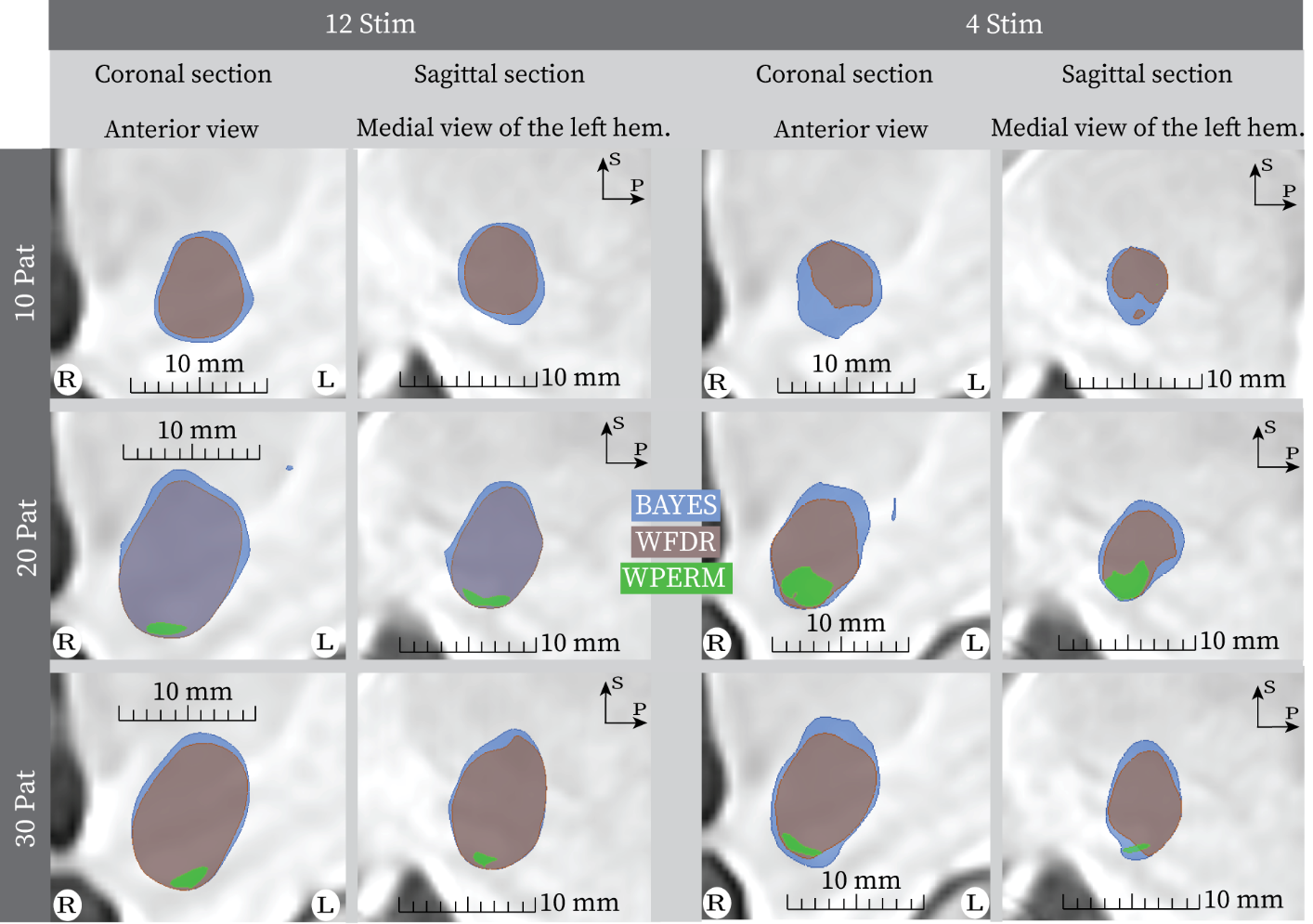


**Supplementary Figure S3**. Examples of obtained PSS in anatomical atlas. The PSS generated by BAYES (blue), WFDR (brown) and WPERM (green) are shown in coronal and sagittal sections. Going from top to bottom the sample size of the PSS was 10, 20 and 30 patients with 12 stimulations each (left columns) and 4 stimulations each (right columns).

# Absolute volumes - PD cohort


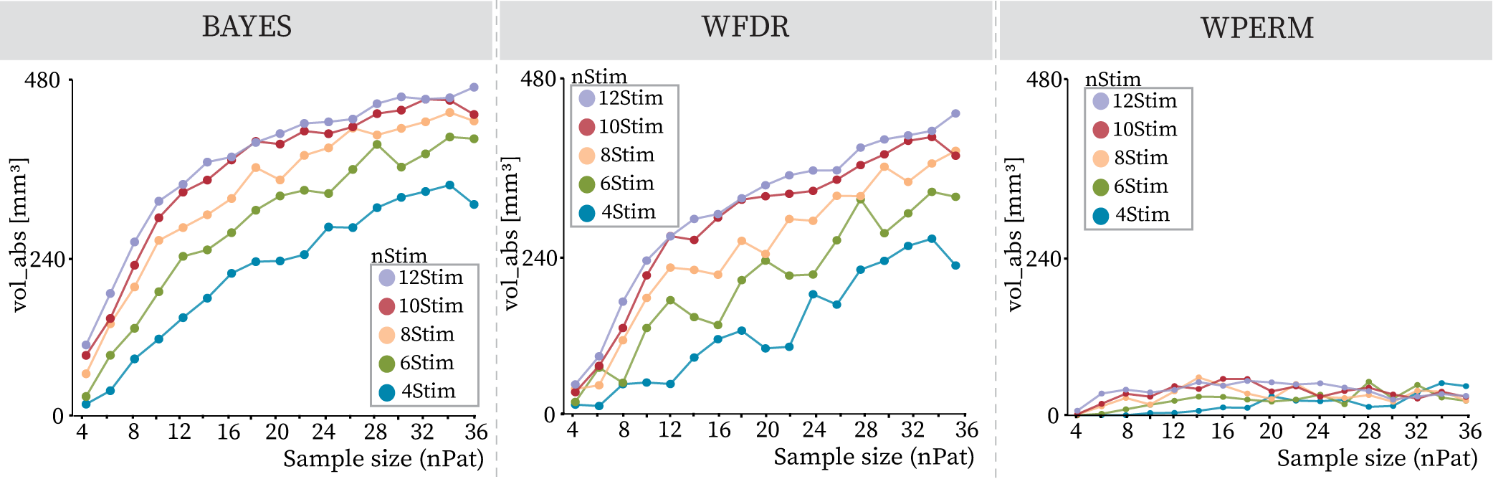


**Supplementary Figure S4.** Absolute PSS volumes in mm^3^ for each statistical test, patient sample size and stimulation count. The dots indicate the mean values among patient samplings for each patient sample size, and the different colors refer to the number of stimulations per patient, as reported in the legend.

# Absolute centroid distances - PD cohort


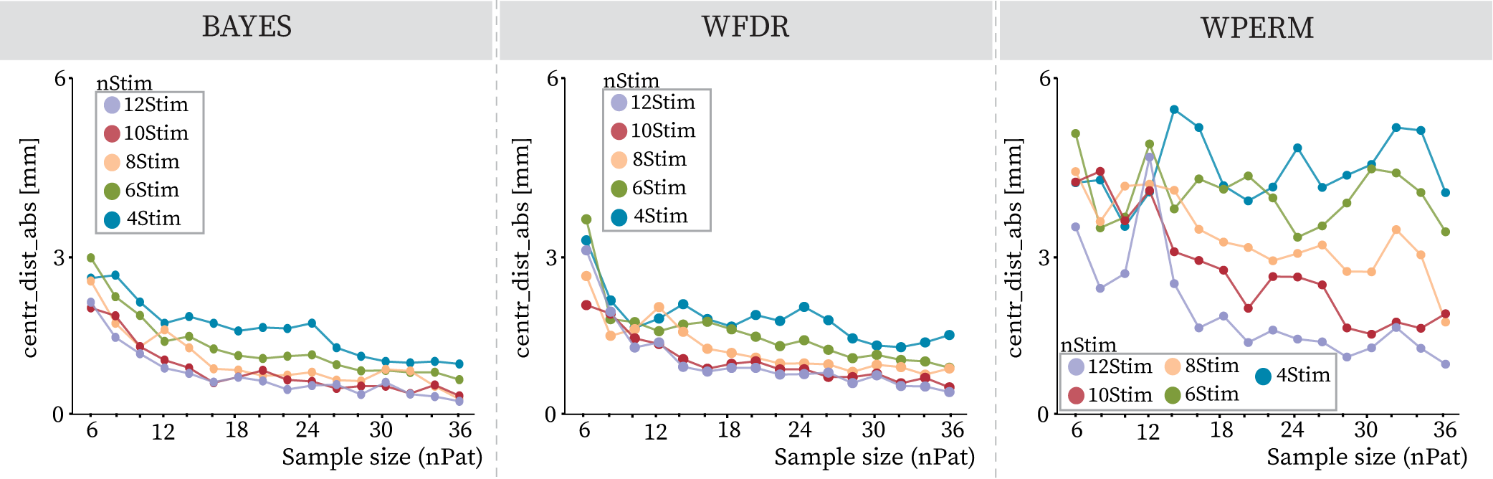


**Supplementary Figure S5.** Absolute centroid distances in mm with increasing sample size (nPat) for different stimulation counts (nStim) and statistical methods. The dots represent average values across patients samplings and the line color refers to the stimulation count as reported in the legend.

# Absolute volumes – ET cohort


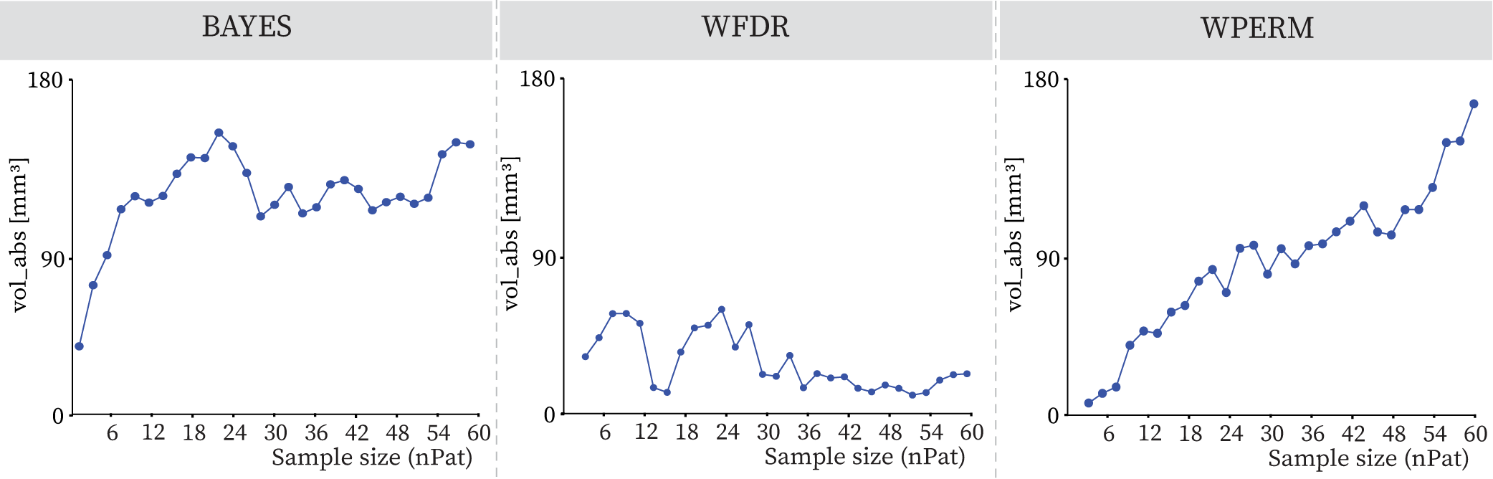


**Supplementary Figure S6.** Absolute PSS volumes in mm^3^ for each statistical test and patient sample size for ET cohort. The dots indicate the mean values among patient samplings for each patient sample size.

# Absolute centroid distances – ET cohort


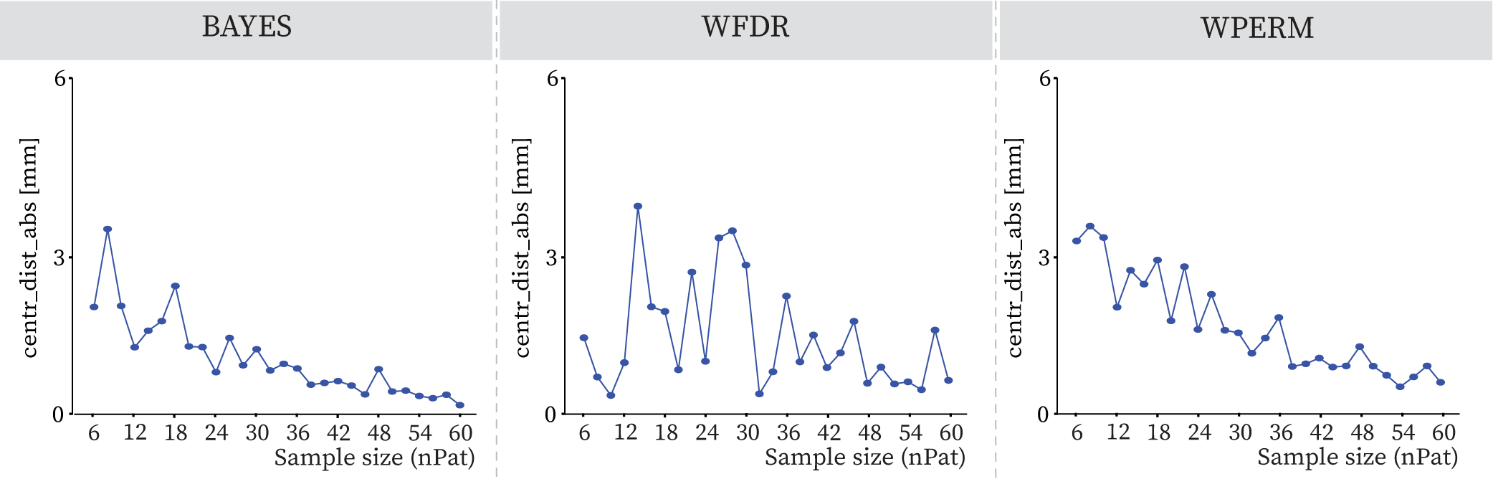


**Supplementary Figure S7.** Absolute centroid distances in mm for each statistical test and patient sample size for the ET cohort. The dots indicate the mean values among patient samplings for each patient sample size.

# Metrics combination – ET cohort


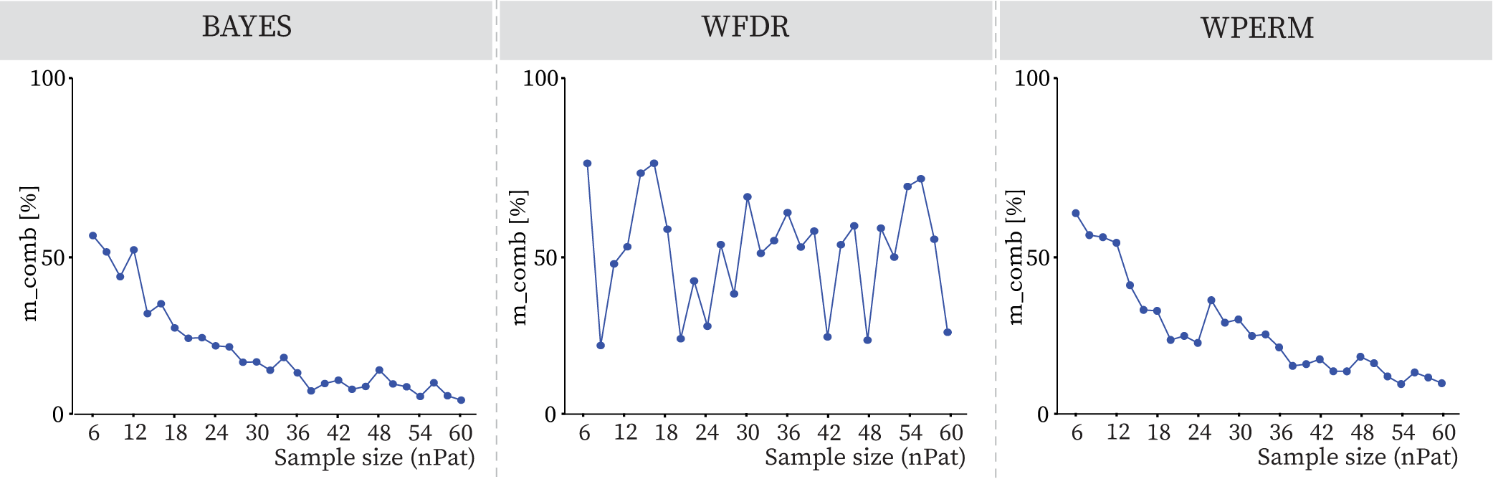


**Supplementary Figure S8.** Metrics combination score for each statistical test and patient sample size for ET cohort. The metrics combination was calculated as mean(vol_diff, (100-dice), centr_dist) where lower values indicate higher stability of the PSS for a certain sample size. The dots indicate the mean values among patient samplings for each patient sample size. Both BAYES and WPERM reached stability at nPat=38, while WFDR did not stabilize.

# Bayesian Logistic Regression model validation

Table SII. Validation results for Bayesian Logistic Regression model. The model was validated with a 70%/30% train/test split for each different statistical method (BAYES, WFDR, WPERM) and metric (centr_dist, dice, vol_diff, all metrics). The model performance was evaluated by calculating the accuracy and F1 score on the test set.

| **Statistical test** | **Metric** | **Accuracy on test** | **F1 on test** |
| --- | --- | --- | --- |
| BAYES | Centr_dist | 0.96 | 0.97 |
| WFDR | Centr_dist | 0.96 | 0.97 |
| WPERM | Centr_dist | 0.92 | 0.83 |
| BAYES | Dice | 0.85 | 0.86 |
| WFDR | Dice | 1.0 | 1.0 |
| WPERM | Dice | 0.73 | 0.63 |
| BAYES | Vol_diff | 0.96 | 0.94 |
| WFDR | Vol_diff | 0.96 | 0.93 |
| WPERM | Vol_diff | 0.96 | 0.20 |
| BAYES | All | 0.92 | 0.93 |
| WFDR | All | 0.97 | 0.97 |
| WPERM | All | 0.87 | 0.68 |

# References

Depaoli, Sarah, Sonja D. Winter, and Marieke Visser. 2020. “The Importance of Prior Sensitivity Analysis in Bayesian Statistics: Demonstrations Using an Interactive Shiny App.” *Frontiers in Psychology* 11 (November): 608045. https://doi.org/10.3389/fpsyg.2020.608045.
